# Aldehyde dehydrogenase 2 inhibits inflammatory response and regulates atherosclerotic plaque

**DOI:** 10.18632/oncotarget.9384

**Published:** 2016-05-15

**Authors:** Chang Pan, Jun-hui Xing, Cheng Zhang, Ying-mei Zhang, Lue-tao Zhang, Shu-jian Wei, Ming-xiang Zhang, Xu-ping Wang, Qiu-huan Yuan, Li Xue, Jia-li Wang, Zhao-qiang Cui, Yun Zhang, Feng Xu, Yu-guo Chen

**Affiliations:** ^1^ Department of Emergency and Chest Pain Center, Qilu Hospital, Shandong University, Ji'nan, China; ^2^ Key Laboratory of Cardiovascular Remodeling and Function Research, Ministry of Education and Ministry of Public Health of People's Republic of China, Qilu Hospital, Shandong University, Ji'nan, China; ^3^ Institute of Emergency and Critical Care Medicine, Qilu Hospital, Shandong University, Ji'nan, China; ^4^ The Key Laboratory of Emergency and Critical Care Medicine affiliated to Health Commission of Shandong Province, Qilu Hospital, Shandong University, Ji'nan, China; ^5^ Department of Cardiology, Qilu Hospital, Shandong University, Ji'nan, China; ^6^ Shanghai Institute of Cardiovascular Diseases, Zhongshan Hospital, Fudan University, Shanghai, China

**Keywords:** ALDH2, polymorphism, atherosclerotic plaque vulnerability, inflammation, MAPK signaling pathway, Pathology Section

## Abstract

Previous studies demonstrated that aldehyde dehydrogenase 2 (ALDH2) rs671 polymorphism, which eliminates ALDH2 activity down to 1%-6%, is a susceptibility gene for coronary disease. Here we investigated the underlying mechanisms based on our prior clinical and experimental studies. Male apoE−/− mice were transfected with GFP, ALDH2-overexpression and ALDH2-RNAi lentivirus respectively (n=20 each) after constrictive collars were placed around the right common carotid arteries. Consequently, ALDH2 gene silencing led to an increased *en face* plaque area, more unstable plaque with heavier accumulation of lipids, more macrophages, less smooth muscle cells and collagen, which were associated with aggravated inflammation. However, ALDH2 overexpression displayed opposing effects. We also found that ALDH2 activity decreased in atherosclerotic plaques of human and aged apoE−/− mice. Moreover, *in vitro* experiments with human umbilical vein endothelial cells further illustrated that, inhibition of ALDH2 activity resulted in elevating inflammatory molecules, an increase of nuclear translocation of NF-κB, and enhanced phosphorylation of NF-κB p65, AP-1 c-Jun, Jun-N terminal kinase and p38 MAPK, while ALDH2 activation could trigger contrary effects. These findings suggested that ALDH2 can influence plaque development and vulnerability, and inflammation via MAPK, NF-κB and AP-1 signaling pathways.

## INTRODUCTION

Acute coronary syndrome (ACS) is the main clinical subtype of atherosclerotic coronary heart disease (CAD) with an increasing morbidity and mortality. It is well known that aldehyde dehydrogenase 2 (ALDH2), as the key enzyme for classical alcohol metabolism, displays an important functional single-nucleotide polymorphism (SNP), or rs671-Glu504Lys polymorphism [1]. Previous studies demonstrated that ALDH2 rs671 mutant displays significantly reduced enzyme activity (1%-6% of wild-type) [2, 3]. This mutation exists in 30%-50% of East Asians, which is ~6% of the world population [1]. The allele occurs in most regions of China, Japan, Korea, Mongolia, and Ecuador with the highest frequency found in Indians of Ecuador Highlands and Southeast Asia. The allele is also present in certain American populations such as Native and Indian Americans although it is rare in Caucasians [4]. Previous studies have shown that the ALDH2 Glu504Lys polymorphism is associated with some drinking-related diseases such as alcoholic cardiomyopathy, alcohol liver cirrhosis and alcohol chronic pancreatitis [5, 6]. Recently, some studies have indicated that ALDH2 and its polymorphisms are involved in ACS and CAD. Most of these work of ALDH2 tended to focus on myocardium, with limited information on the vasculature. Several surveys, including studies conducted in Japan, China and Korea, have indicated that the ALDH2 Glu504Lys polymorphism is closely associated with myocardial infarction and/or ACS in East Asians and the ALDH2 mutant genotypes (*1/*2 and *2/*2) are considered independent risk factors of ACS [7–9]. In 2012, a genome-wide association study of common SNPs identified that ALDH2 is a CAD susceptibility gene [10]. However, the exact molecular mechanisms underlying how ALDH2 affects the occurrence of ACS or CAD remain unclear.

One earlier study from our group demonstrated that ALDH2 mutation is associated with decreased high-density lipoprotein cholesterol (HDL-C) levels [11], suggesting that ALDH2 might influence atherosclerosis progression and subsequently CAD via lipid accumulation. Our data found that ox-LDL may decrease ALDH2 dehydrogenase activity obviously, although inhibition of Poly(ADP-ribose) polymerase restores the ALDH2 dehydrogenase activity by preventing expression of SIRT3 and maintaining the acetylation level of ALDH2 [12]. Moreover, one of our studies displayed that ALDH2 activation with hyperacetylation by SIRT3 inactivation improves human aortic endothelial function [13]. Furthermore, we also found that ALDH2 inhibition by hyperglycemia aggravates mitochondrial impairment, which is essential to cellular energy metabolism [14]. Recently, our studies further indicated that the ALDH2 mutation is tied with levels of high-sensitivity C-reactive protein, a classical inflammatory biomarker, and the number of circulating endothelial progenitor cells [15]. In addition, previous studies showed that overexpression of ALDH2 attenuates myocardial apoptosis [16] and ALDH2 plays an important role in myocardial protection [17–19]. It is well conceived that lipids, endothelial dysfunction or injury-repair imbalance, and inflammation play critical roles in atherosclerosis progression and atherosclerotic plaque vulnerability, which are recognized as a pivotal mechanism of ACS [20]. Furthermore, inflammation, which can be triggered by oxidative stress, is the main cause of many human diseases, including cardiovascular diseases [21]. Therefore, we hypothesized that ALDH2 might play a role in vasculature by the way of influencing plaque development, vulnerability and inflammation, leading to acute coronary events. This hypothesis is further supported by recent reports which indicated that ALDH2 with different activities has different impacts on oxidative stress and subsequent inflammation. Recent studies found that functional ALDH2 could metabolize aldehydes, such as the endogenous oxidative stressor 4-hydroxy-2-nonenal (4-HNE), into much less reactive chemical species [22]. Our recent study also revealed that improvement in ALDH2 activity is capable of ameliorating oxidative stress [23].

In the present study, to investigate the effect of ALDH2 on vasculature, we hypothesized that ALDH2 could affect plaque development, stability and inflammation. To validate this hypothesis and investigate the underlying signaling pathways, a series of *in vivo* and *in vitro* experiments were designed and performed.

## RESULTS

### ALDH2 expression and activity in mice

**Figure 1:**
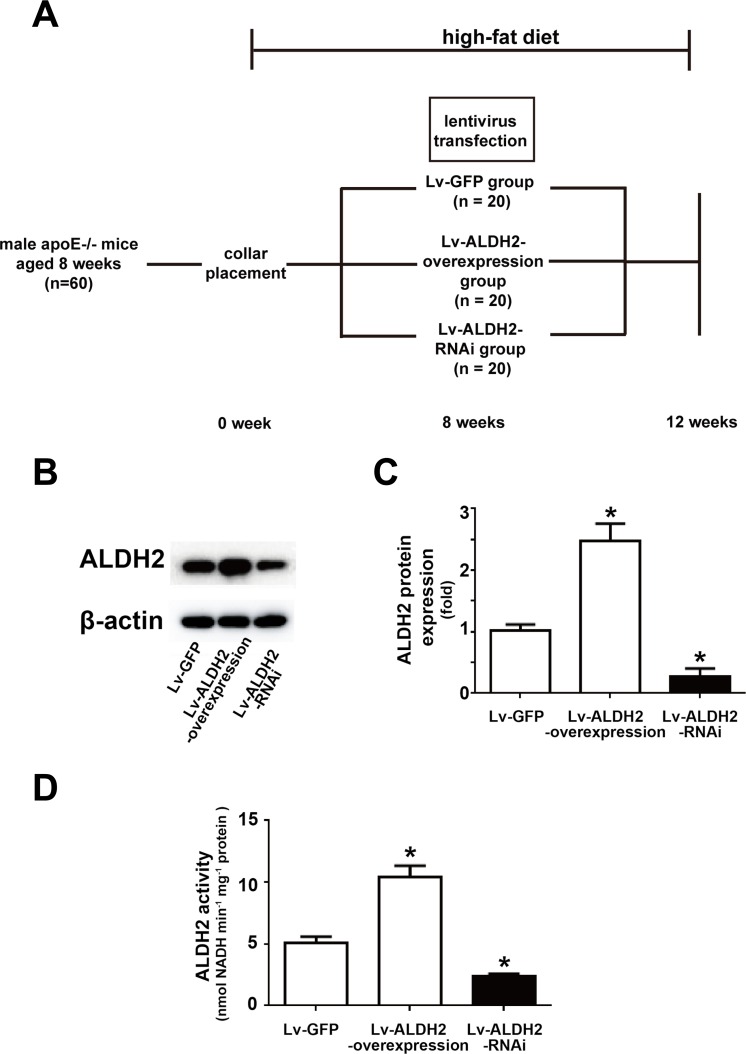
Time line, grouping and ALDH2 expression after lentivirus transfection in apoE−/− mice **A.** Time line and grouping of the mice experiment. **B.** Representative blot of ALDH2 protein expression in three groups of the apoE−/− mice. **C.** Statistical analysis of relative ALDH2 expression in three groups. **D.** Histograms of ALDH2 activity in three groups. All data were showed as mean±SEM. *: *P* < 0.05 *vs* Lv-GFP group.

All mice were healthy in the process of collar placement and lentivirus treatment (Figure 1A), but one mouse in the Lv-ALDH2-RNAi group and one mouse in the Lv-ALDH2-overexpression group were lost before the day of sacrifice. To estimate the efficiency of lentivirus-mediated gene overexpression and knockdown *in vivo*, the levels of ALDH2 protein expression in carotid plaques and ALDH2 activity of aortas were detected (Figure 1B-1D). Compared with the Lv-green fluorescent protein (GFP) group, ALDH2 protein expression levels in the Lv-ALDH2-overexpression group and Lv-ALDH2-RNAi group were increased by approximately 150% and decreased by 80%, respectively (both P < 0.05, Figure 1B and 1C). Moreover, in accordance with protein expression levels in different groups, the activity of ALDH2 in the Lv-ALDH2-overexpression group was increased but decreased in the Lv-ALDH2-RNAi group (both P < 0.05, Figure 1D).

### Effect of ALDH2 on body weight and serum lipid levels

For each group of mice, body weight and the serum concentrations of lipid were measured at the end of the experiment. Neither the carotid collar placement nor the lentivirus injection had effects on their body weight (Table S1). There were also no significant differences of serum levels of total cholesterol, triglyceride, low-density lipoprotein cholesterol (LDL-C) and HDL-C among the Lv-ALDH2-overexpression group, the Lv-ALDH2-RNAi group and the Lv-GFP group (Table S2), suggesting that ALDH2 gene transfer has little effects on serum lipid levels.

### Effect of ALDH2 on the extent of aortic atherosclerosis

**Figure 2:**
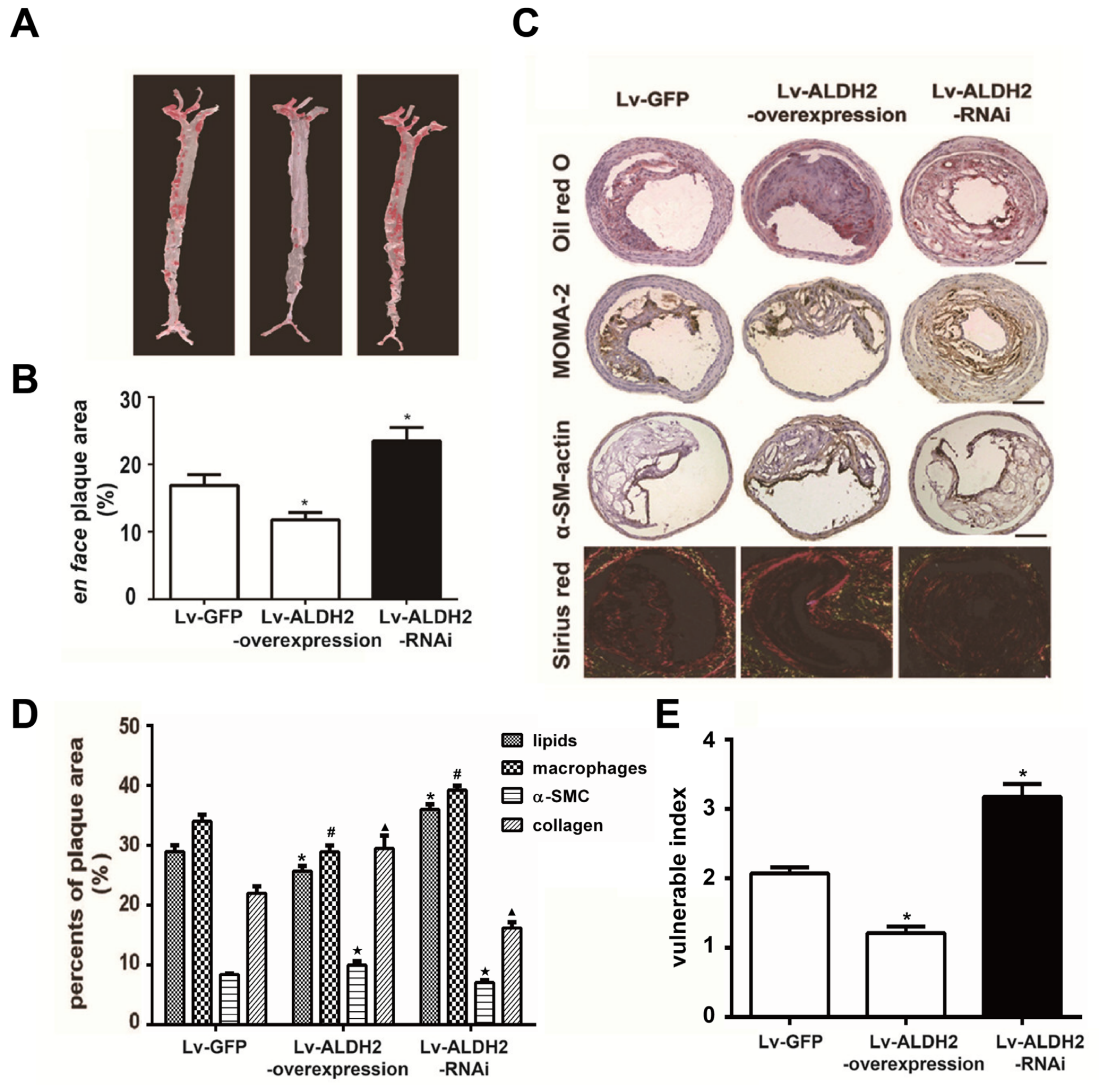
Changes of atherosclerotic plaques in aortas and carotid plaques composition in apoE−/− mice after lentivirus transfection **A.** Representative *en face* photographs of aortas in three groups showing oil red O-stained atherosclerotic plaques. **B.** Quantitative measurement of *en face* plaque area (%) in the whole aorta. *: *P* < 0.05 *vs* Lv-GFP group. **C.** Representative Oil-red O staining, MOMA-2 immunostaining, α-SM-actin immunostaining and Sirius-red staining in three groups of the apoE−/− mice; the bars represent 100μm. **D.** Column graph of quantification of staining results in Figure 2C by Image-Pro Plus 6.0 Software. *, ^#^, ★, ^▴^: *P* < 0.05, percent of lipids, macrophages, α-SMCs and collagen compared with Lv-GFP group, respectively. All data were presented as mean±SEM. **E.** Bar chart of vulnerable index in three groups of the apoE−/− mice. The vulnerability indexs in the Lv-GFP, Lv-ALDH2-overexpression and Lv-ALDH2-RNAi groups are 2.10±0.10, 1.40±0.10 and 3.20±0.19, respectively. *: *P* < 0.05 *vs* Lv-GFP group.

To evaluate the effect of ALDH2 on the extent of aortic atherosclerosis, total lesion area of aortas were measured using oil-red O staining at 4 weeks after lentivirus transfection. The extent of atherosclerosis in Lv-GFP group was intermediate between that observed in the Lv-ALDH2-overexpression and Lv-ALDH2-RNAi groups. Alternatively, Lv-ALDH2-overexpression group showed significantly reduced *en face* plaque area compared with the Lv-GFP group (11.80%±1.10% vs 16.92%±1.55%, P < 0.05), while the Lv-ALDH2-RNAi group had increased atherosclerotic lesion areas (23.51%±1.99%, P < 0.05, Figure 2A and 2B).

### Effect of ALDH2 on component changes of carotid plaques

The relative content of lipids, macrophages, α-smooth muscle cells (SMCs) and collagen in the plaques was measured by histological and immunohistochemical staining (Figure 2C). In Lv-ALDH2-overexpression group, the ratios of lipids and macrophages were lower in carotid plaques, whereas α-SMCs and collagen ratios were higher compared with the Lv-GFP group (P < 0.05). However, plaques in the Lv-ALDH2-RNAi group displayed a higher content of lipids and macrophages, but a lower content of α-SMCs and collagen (P < 0.05, Figure 2C and 2D). These led to a change of the plaque vulnerability index. Compared with the Lv-GFP group, the vulnerability index in the Lv-ALDH2-overexpression group is lower, but the vulnerability index in the Lv-ALDH2-RNAi group is higher (P < 0.05, Figure 2E).

### Effect of ALDH2 on the expression of inflammation markers in carotid plaques

**Figure 3:**
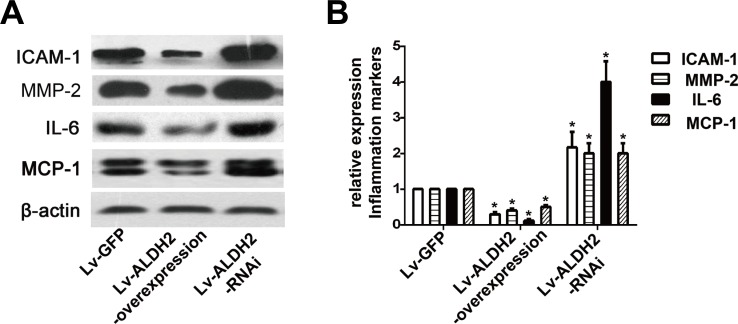
Protein expression of inflammatory cytokines in three groups of the apoE−/− mice after lentivirus transfection **A.** Representative western images of ICAM-1, IL-6, MMP-2 and MCP-1 in three groups. **B.** Statistical analysis of the data presented in Figure 3A. The data were presented as mean±SEM. *: *P* < 0.05 *vs* Lv-GFP group.

The protein expression levels of intercellular adhesion molecule-1 (ICAM-1), matrix metalloproteinase-2 (MMP-2), interleukin-6 (IL-6) and monocyte chemotactic protein-1 (MCP-1) were detected by western blot (Figure 3). Compared with the Lv-GFP group, the expression of ICAM-1, MMP-2, IL-6 and MCP-1 were reduced approximately by 70%, 50%, 80% and 50% in the Lv-ALDH2-overexpression group but increased approximately by 100%, 90%, 300% and 100% in the Lv-ALDH2-RNAi group (P < 0.05), respectively.

### ALDH2 expression and activity in atherosclerotic plaques

**Figure 4:**
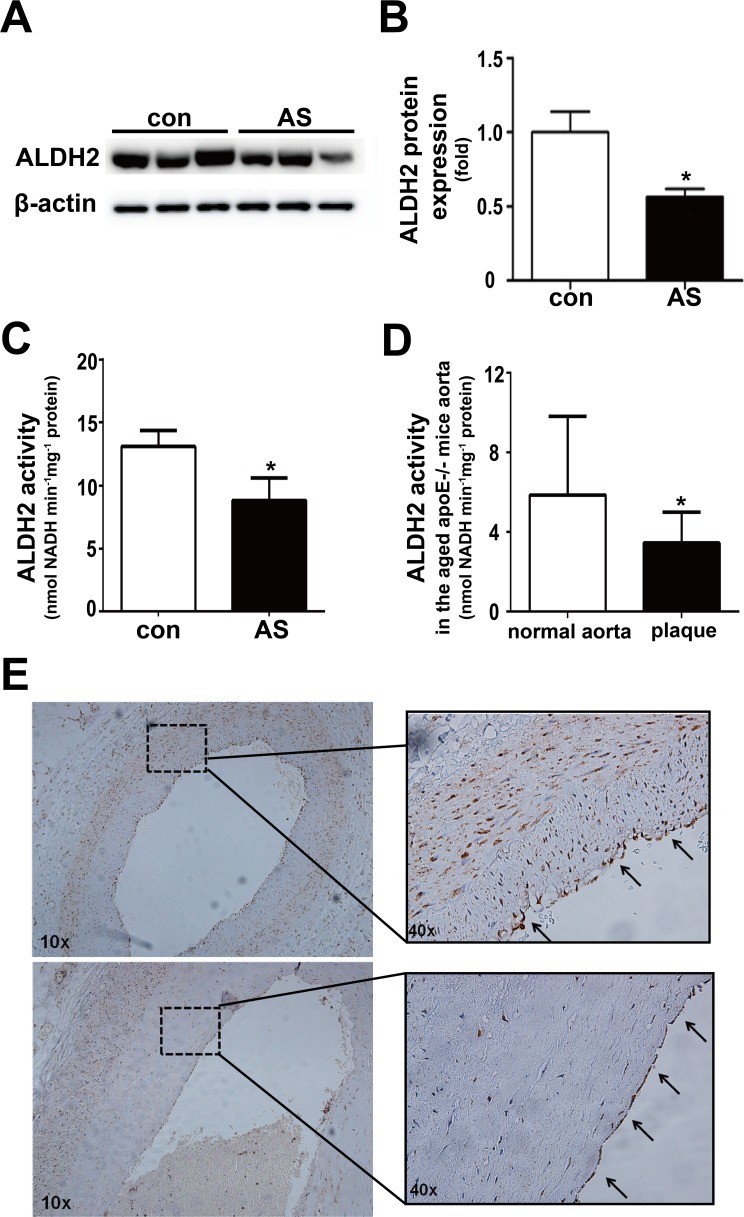
Expression and activity of ALDH2 in the vessels with or without atherosclerotic plaques **A.** Representative blot of ALDH2 protein expression in human normal coronary arteries and atherosclerotic vessels. **B.** Statistical analysis of the data presented in Figure 4A. **C.** Histogram of ALDH2 activity in human normal coronary arteries and atherosclerotic vessels. **D.** Histogram of ALDH2 activity of old-aged apoE−/− mice aortas. **E.** Representative ALDH2 immunostaining in human coronary arteries. The arrows show that ALDH2 is expressed in endothelial cells. Con: normal coronary arteries, AS: atherosclerotic vessels. *: *P* < 0.05 *vs* control. The data were presented as mean±SEM.

We further detected ALDH2 expression and activity in postmortem human coronary arteries. Compared with human coronary arteries without plaques, both ALDH2 expression and activity in the atherosclerotic vessels were decreased (both P < 0.05) (Figure 4A-4C). Similarly, ALDH2 activity in the aged apoE−/− mouse aortas with plaques was also lower than that in normal aortas (Figure 4D). In addition, we found that ALDH2 was expressed in endothelial cells and SMCs in human coronary arteries (Figure 4E).

### Time- and dose-dependent effects of LPS on expression of MCP-1 and ICAM-1 protein in HUVECs

To select the optimal concentration and incubation time for lipopolysaccharide (LPS), expression of MCP-1 and ICAM-1 protein was measured in human umbilical vein endothelial cells (HUVECs) exposed to LPS at various concentrations for 12 hours and 100ng/ml LPS for various times. High concentrations and long LPS incubation times resulted in significant MCP-1 and ICAM-1 production (Figure S1). Thus, we chose 100ng/ml LPS and a 12-hour HUVEC incubation time.

### Effects of ALDH2 on the expression of MCP-1 and ICAM-1 protein in HUVECs activated with LPS

**Figure 5:**
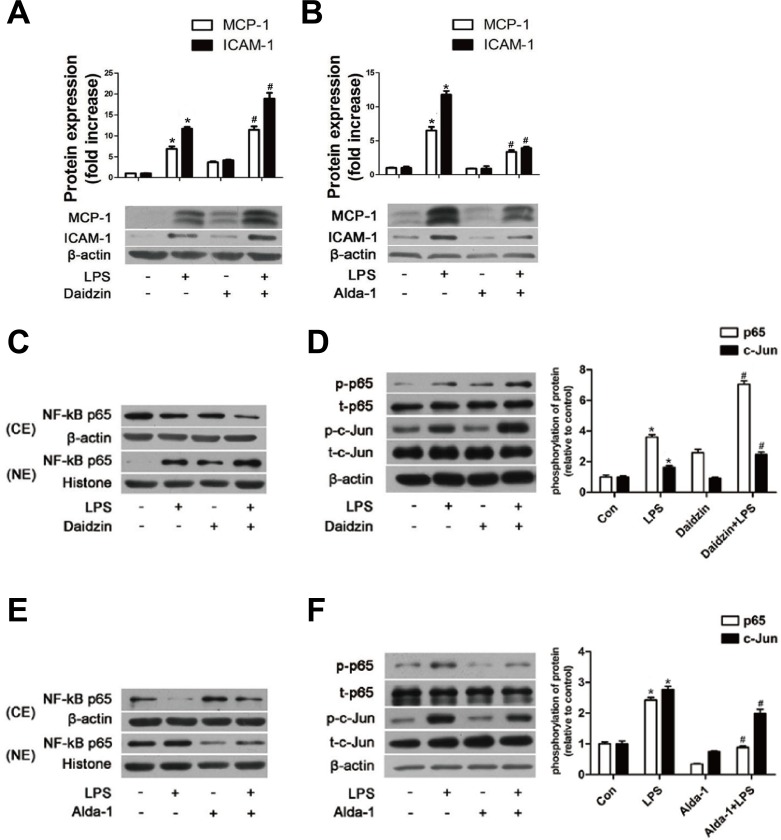
The effects of ALDH2 on MCP-1, ICAM-1, and NF-κB and AP-1 transcription factors in HUVECs **A.** MCP-1 and ICAM-1 expression in HUVECs pre-treated with Daidzin before LPS treatment. **B.** MCP-1 and ICAM-1 expression in HUVECs pre-treated with Alda-1 before LPS treatment. Values are expressed as means±SEM from three independent experiments. *: *P* < 0.05 *vs* control. ^#^: *P* < 0.05 *vs* LPS treatment alone. **C.** NF-κB P65 protein expression in HUVECs pre-treated with or without Daidzin before LPS treatment. **D.** Phosphorylation of NF-κB P65 and AP-1 c-Jun in HUVECs with or without Daidzin before LPS treatment. **E.** Protein expression of NF-κB P65 in HUVECs treated with or without Alda-1 before LPS treatment. **F.** Phosphorylation levels of NF-κB P65 and AP-1 c-Jun in HUVECs with or without Alda-1 before LPS treatment. Values are expressed as means±SEM from three independent experiments. *: *P* < 0.05 *vs* control. ^#^: *P* < 0.05 *vs* LPS treatment alone.

We first used daidzin, a known potent and selective inhibitor of ALDH2 [24], to inhibit ALDH2 activity. Daidzin (25μM) reduced ALDH2 activity by approximately 50% with little effect from the solvent DMSO (Figure S2A). As shown in Figure 5A, both MCP-1 and ICAM-1 protein were increased after inhibiting ALDH2 activity. To determine the exact effect of ALDH2, we used Alda-1, a novel small molecule which can activate catalysis for both wild-type ALDH2 (ALDH2*1) and ALDH2*2 [25, 26], to stimulate ALDH2 activity. We demonstrated that 20μM Alda-1 increased ALDH2 activity approximately by 80% with no effect from the solvent DMSO (Figure S2B). As shown in Figure 5B, Alda-1 significantly inhibited the expression of MCP-1 and ICAM-1 proteins.

### Effects of ALDH2 on the expression of NF-κB and AP-1 transcription factors

Nuclear factor-κB (NF-κB) is a pivotal regulator of inflammation, and activation of NF-kB is required to induce expression of MCP-1 and ICAM-1 in LPS-activated endothelial cells [27]. The location of NF-κB subunit P65 was determined using western blot. LPS at 100ng/ml triggered NF-κB p65 subunit translocation from the cytoplasm to the nucleus. HUVECs pretreated with daidzin displayed an increase of nuclear translocation (Figure 5C), and Alda-1 inhibited the nuclear translocation (Figure 5E). In addition, the phosphorylation of NF-κB p65^S536^, as a type of posttranslational modification, is reported to contribute to the activity of NF-κB p65 and prolong the activity of NF-κB in the nucleus [28]. As shown in Figure 5D, the phosphorylation of NF-κB p65^S536^ was increased in LPS-treated HUVECs pretreated with daidzin compared with that in HUVECs treated with LPS alone, whereas it was decreased in Alda-1-treated HUVECs (Figure 5F). Increasing evidence indicates the importance of transcription factor activator protein-1 (AP-1) independent of NF-κB in the pathogeneses of endothelial inflammation. The phosphorylation of AP-1 c-Jun^ser73^ could enhance its transactivation potential. In Figure 5D, the phosphorylation of AP-1 c-Jun^S73^ was enhanced in LPS-treated HUVECs pretreated with daidzin similar to NF-κB P65; however, it was reduced in Alda-1-treated HUVECs (Figure 5F). In addition, ALDH2 had little effect on the expression of AP-1 c-Jun protein.

### Effects of ALDH2 on the expression of the MAPK system

**Figure 6:**
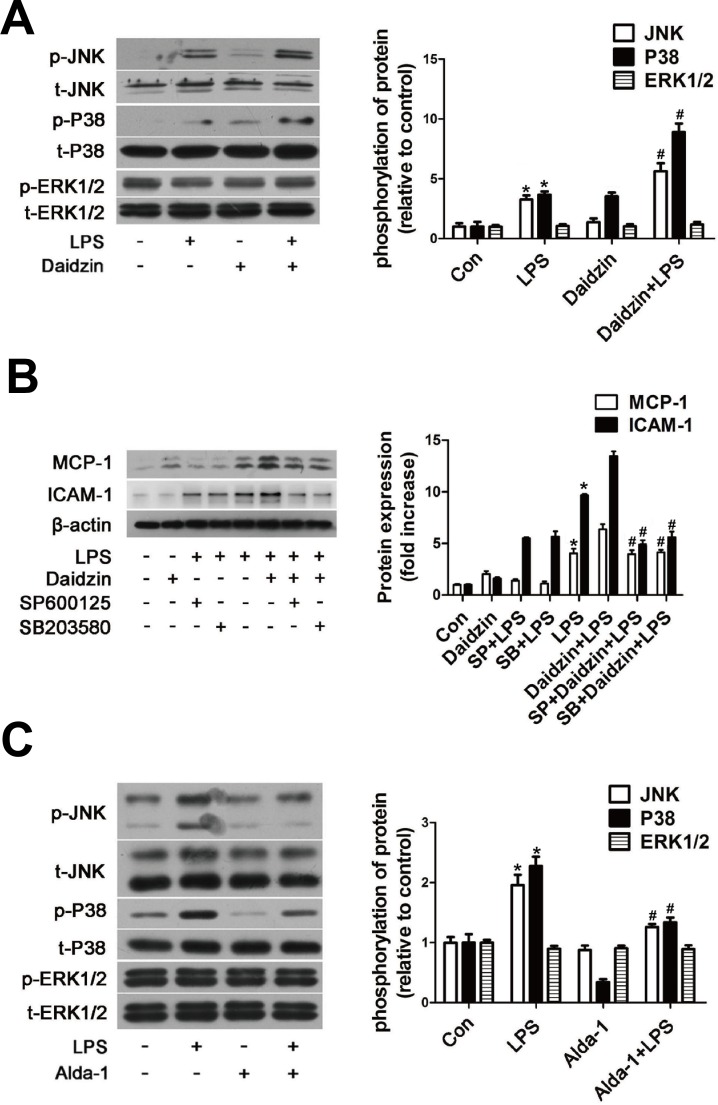
ALDH2 regulates expression of MCP-1 and ICAM-1 protein *via* JNK and P38 MAPK system in LPS-activated HUVECs **A.** Phosphorylation of JNK, p38 MAPK and ERK1/2 in HUVECs stimulated by Daidzin before LPS treatment. *: *P* < 0.05 *vs* control. ^#^: *P* < 0.05 *vs* LPS treatment alone. **B.** MCP-1 and ICAM-1 expression after SP600125 (20μm) or SB203580 (20μm) treatment before LPS stimulation in HUVECs. Con: control; SP600125: JNK inhibitor; SB203580: P38 inhibitor. *: *P* < 0.05 *vs* control. ^#^: *P* < 0.05 *vs* LPS+Daidzin treatment. **C.** Phosphorylation of JNK, p38 MAPK and ERK1/2 in HUVECs stimulated by Alda-1 before LPS treatment. *: *P* < 0.05 *vs* control. ^#^: *P* < 0.05 *vs* LPS treatment alone. All values are expressed as means±SEM from three independent experiments.

Mitogen-activated protein kinase (MAPK) cascade, which can be upstream of NF-κB and AP-1, accounts for the cellular injury mechanisms during various stresses, including inflammation response. Next, we analyzed whether such a cascade was responsible for the regulation of MCP-1 and ICAM-1 expression by ALDH2. Western blot results revealed that phosphorylation of Jun-N terminal kinase (JNK) and p38 MAPK but not extracellular signal-regulated kinase 1/2 (ERK1/2) were further enhanced in LPS-activated HUVECs pretreated with daidzin compared with that in HUVECs treated with LPS alone (Figure 6A). Moreover, we confirmed that 20μM SP600125 (JNK inhibitor) and 20μM SB203580 (p38 MAPK inhibitor) preferentially inhibited expression of MCP-1 and ICAM-1 protein in LPS-activated HUVECs pretreated with daidzin compared with HUVECs treated with LPS alone (Figure 6B). As shown in Figure 6C, Alda-1 inhibited phosphorylation of JNK and p38 MAPK but not ERK1/2.

## DISCUSSION

The major findings of our present study were that ALDH2 overexpression in the vulnerable carotid plaque of apoE−/− mice led to higher ALDH2 activity and a more stable plaque with less accumulation of lipids, fewer macrophages, more SMCs and collagen, as well as with reduced inflammatory factors. However, gene silencing of ALDH2 displayed opposing effects. *In vitro* experiments demonstrated that ALDH2 influenced inflammatory molecules including MCP-1 and ICAM-1 through some members of the MAPK family (JNK and p38 MAPK, but not ERK1/2) and the NF-κB and AP-1 signaling pathways. To the best of our knowledge, this is the first report that ALDH2 can directly influence plaque development, stability and inflammation through MAPK, NF-κB and AP-1 signaling pathways, which suggests an important explanation for ALDH2 functioning as a CAD or ACS susceptibility gene and supplies solid evidence for ALDH2 effects on vasculature.

Previous pathological surveys in human have demonstrated that components of plaques rather than size of plaques play a more prominent role in the development of ACS, as its major cause is atherosclerotic plaque disruption, which may result from increased plaque vulnerability [29]. Plaque vulnerable to disruption is characterized by increased content of lipids and macrophages and reduced content of collagen and α-SMCs [30]. To evaluate vulnerable plaques histologically, Shiomi put forward the “plaque vulnerability index”, which incorporates the cumulative effects of the known morphological risk factors [31]. Our present data revealed that the Lv-ALDH2-overexpression group exhibited higher ALDH2 activity, reduced lesion area, less accumulation of lipids and macrophages, more collagen and SMCs in atherosclerotic plaques, with the Lv-ALDH2-RNAi exhibiting opposite effects. These findings suggest a key role for ALDH2 in governing components of atherosclerosis. Furthermore, this study demonstrated that all component changes of plaques in the Lv-ALDH2-overexpression group contributed to a decreased plaque vulnerability index, whereas those components of the Lv-ALDH2-RNAi group resulted in a much larger index. These results suggest that ALDH2 gene overexpression or ALDH2 with higher activity could enhance atherosclerotic plaque stability, whereas ALDH2 gene knockdown might promote plaque vulnerability. In addition, we found that there was no significant difference of HDL-C levels among the three groups, which seems that there was a discrepancy with previous human studies. In fact, according to previous studies, whether ALDH2 can influence serum lipids in human is still a controversial and uncertain issue, which should be still elucidated with studies in larger population sample in the future [8, 15, 32, 33]. Besides, the species difference between humans and mice may make a contribution to the discrepancy.

Existing evidence indicates that inflammation plays an important role in plaque instability. IL-6, MMP-2, ICAM-1 and MCP-1 are significant markers of inflammation and important inflammatory biomarkers to identify vulnerable plaques. In our present experiment, we observed that changes of expression of IL-6, MMP-2, ICAM-1 and MCP-1 were negatively correlated with expression of ALDH2 protein. The protein levels of IL-6, MMP-2, ICAM-1 and MCP-1 were reduced in the Lv-ALDH2-overexpression group. Changes of these cytokines were simultaneous with a more stable plaque phenotype as manifested by increased collagen and α-SMCs and decreased macrophages and lipids in the carotid plaques. However, the expression of these proteins was increased in the Lv-ALDH2-RNAi group, accompanying a less stable plaque. These results might suggest that ALDH2 may affect the components of plaques and plaque stability via inflammation.

In the present study, we also examined the relationship between ALDH2 and inflammation in HUVECs treated with LPS *in vitro*. Our findings suggest that levels of two important inflammatory molecules, ICAM-1 and MCP-1, could be regulated by ALDH2, which was consistent with the *in vivo* results.

Among the known cell-signaling molecules mediating inflammation, NF-κB and AP-1 are known to initiate the production of various inflammatory cytokines [34, 35]. IκB degradation through proteolysis and phosphorylation of NF-κB p65^S536^ contribute to the activity of NF-κB [28]. In this study, we observed that the NF-κB p65 levels in the nuclei of HUVECs and its phosphorylation induced by LPS were increased by ALDH2 inhibition but reduced by ALDH2 activation, consistent with the previous notion of NF-κB in triggering inflammation. These results suggested that the effect of ALDH2 on the expression of ICAM-1 and MCP-1 was at least partly mediated by the regulation of NF-κB activity in HUVECs in response to LPS. Another nuclear transcription factor, AP-1, which has enhanced activity following phosphorylation, could bind to the upstream promoters of inflammatory cytokines to induce inflammation [36]. In this study, we observed that the phosphorylation level of AP-1 c-Jun induced by LPS was increased by ALDH2 inhibition and reduced by ALDH2 activation. These results clearly demonstrated that the mitochondrial ALDH2 significantly regulates NF-κB and AP-1 activity, and indicated that the effect of ALDH2 on the expression of ICAM-1 and MCP-1 was at least partly mediated by the regulation of NF-κB and AP-1 activity in HUVECs in response to LPS.

MAPK signaling, including JNK, ERK1/2 and p38 MAPK, which can be activated by LPS and TNF-α, is reported to play an important role in LPS-induced inflammation [37]. We assessed the effect of ALDH2 on LPS-induced phosphorylation of MAPKs. LPS-treated HUVECs exhibited increased phosphorylation levels of JNK and p38 MAPK, and both SP600125 and SB203580 reduced the expression of ICAM-1 and MCP-1. These results were consistent with previous studies [37]. These results revealed the critical roles of JNK and p38 MAPK in LPS-induced inflammation in HUVECs. Furthermore, our study demonstrated that phosphorylation of JNK and p38 MAPK was reduced by ALDH2 activation and enhanced by ALDH2 inhibition. Up to date, the detailed and precise mechanism underlying how ALDH2 inhibits JNK/p38-NF-κB signaling pathway has not been fully established. This may be related to the clearance of toxic aldehydes by ALDH2 [38]. 4-HNE induces phosphorylation of JNK and activates it through down-regulation of HSP70, while ALDH2 overexpression detoxifies 4-HNE, leading to inhibited JNK phosphorylation. Moreover, acetaldehyde can induce p38 and JNK phosphorylation, while ALDH2 is the key enzyme for acetaldehyde detoxification [39, 40]. These JNK phosphorylation results are consistent with changes in the JNK downstream nuclear signaling AP-1 phosphorylation following LPS treatment in HUVECs. Importantly, elevated ICAM-1 and MCP-1 induced by ALDH2 inhibition in LPS-treated HUVECs were partly abolished by pretreatment of HUVECs with SP600125 and SB203580, suggesting that the JNK and p38 MAPK pathways are functionally involved in the regulation by ALDH2 of the expression of ICAM-1 and MCP-1 in LPS-activated HUVECs. On the other hand, the responses of SP600125 and SB203580 also revealed potential contributions of other signaling pathways in the effects of ALDH2 on inflammation.

Our data here for the first time provide convincing evidence that ALDH2 can influence atherosclerotic plaque development and vulnerability, and inflammatory response via the MAPK, NF-κB and AP-1 signaling pathways. These results are potentially translational. Previous studies showed that continuous usage of some medications in CAD patients, such as nitrates, may drastically inhibit ALDH2 activity [41, 42]. Thus, clinical genotyping when necessary may offer an efficient way to promote early screening, personalized prevention and treatment, ultimately clinical guidance for this huge population, ~6% of the world. These are particularly important during the era of personalized medicine. For susceptible population carrying ALDH2*504Lys allele, prevention and treatment of atherosclerotic diseases, such as CAD, may be optimized with cautious usage of certain medications including nitrates and the addition of drugs or life style modification (such as moderate drinking) to enhance ALDH2 activity while inhibiting inflammation [13, 41].

However, exactly how ALDH2 genetic profile in humans affects the clinical management of ACS remains elusive. Large scale multi-centered RCT studies are still warranted to understand the interplay between ALDH2 polymorphism and atherosclerosis. In addition, it is unrealistic to employee the histopathological techniques used in animal study for the detection of plaque formation in human subjects, making the clinical value of ALDH2 polymorphism more challenging for early diagnosis of atherosclerotic plaque formation using intravascular imaging techniques, such as intravascular ultrasound and optical coherence tomography. On the other hand, our limitation is that our findings from Lv-ALDH2-RNAi mice model can't directly reflect the function of ALDH2 rs671 mutant, therefore gene (wild-type and mutation) knock-in approach should be used to confirm our findings in the future studies.

## MATERIALS AND METHODS

### Reagents and antibodies

HUVEC medium was purchased from ScienCell (Carlsbad, CA). LPS, daidzin and oil-red O were purchased from Sigma (St. Louis, MO). Alda-1 was bought from Tocris Bioscience (USA). Sirius red was bought from Biohao biological technology (China). Opti-MEM Medium and trypsin containing 0.25% EDTA were purchased from Gibco (Rockville, MD). Anti-ICAM-1 antibody and anti-ALDH2 antibody for western blot were obtained from Santa Cruz Biotechnology (Santa Cruz, CA). Anti-ALDH2 antibody for immunohistochemistry was bought from Proteintech (Chicago, USA). Anti-MCP-1, anti-MOMA-2, anti-IL-6, anti-MMP-2 and anti-alpha-smooth muscle actin antibodies were purchased from Abcam (San Francisco, CA). Antibodies specific to JNK, phospho-JNK^Thr-183/Tyr-185^, ERK1/2, phospho-ERK1/2^Thr-202/Tyr-204^, P38 MAPK, phospho-P38 MAPK^Thr-180/Tyr-182^, c-Jun, phospho-c-Jun^Ser-73^ and β-actin were obtained from Cell Signaling Technology (Danvers, MA). Anti-NF-κB P65, anti-phospho-NF-κB P65^Ser536^ antibodies and SP600125 and SB203580 were purchased from the Beyotime Institute of Biotechnology (China). Secondary antibodies used for immunohistochemistry were bought from ZSGB biological technology (China). The constrictive collar (length-2.0 mm, inner diameter-0.30 mm, outer diameter-0.50 mm) was purchased from Shandong Key Laboratory of Medical Polymer Materials (China). The lentivirus vector ALDH2 construct was acquired from Genechem (China).

### Animal model

Sixty male apoE−/− mice aged 8 weeks were purchased from the Beijing University Animal Research Center (Beijing, China). They were kept on a fixed 12 hour light/dark cycle at a constant temperature of 21.0±1.0°C with sufficient food and water. After anesthesia via an intraperitoneal injection of pentobarbital sodium (50mg/kg), a constrictive collar was placed around the right common carotid artery [43], and then the mice were fed a high-fat diet (15% cocoa butter and 0.25% cholesterol). Eight weeks later, the mice were divided into three groups: Lv-GFP group, Lv-ALDH2-overexpression group and Lv-ALDH2-RNAi group (n = 20 each). Then, the lentiviral suspension of GFP, ALDH2 and ALDH2 siRNA was injected by cauda veins of the mice. Moreover, old-aged apoE−/− mice were used for ALDH2 activity detection. All animal procedures were in compliance with the NIH Guide and approved by the Animal Use and Care Committee of Shandong University.

### Serum lipid levels

At the end of the experiment, the mice were fasted overnight and blood samples were collected. The serum concentrations of total cholesterol, triglyceride, LDL-C and HDL-C were measured.

### Histopathological and immunohistochemical staining

In all mice, the right common carotid arteries were collected for histopathological and immunohistochemical analysis. After being fixed with 4% formaldehyde (pH 7.2) overnight at 4°C, and embedded in OCT compound, the t issues samples from the right common carotid arteries were cross-sectioned into 7μm thick pieces at 50μm intervals. These sections were then stained with sirius red for collagen components and oil-red O for lipids, and immunohistochemical staining was used to analyze macrophages and α-SMCs with specific antibodies. All sections were analyzed with an automated image analysis system, Image-Pro Plus 6.0 (Media Cybernetics, USA), for quantitative measurements. The vulnerability index was calculated according to the following formula: positive staining area of (macrophages + lipid) / positive staining area of (α-SMCs + collagen) [31]. Moreover, in order to quantify the extent of atherosclerotic lesions, the whole length of the aorta was excised for quantification of the *en face* plaque area. In brief, the aortas were opened longitudinally after carefully removing adventitial tissue, and stained with Oil red O. The stereomicroscope was uesd to obtain the *en face* images, which were analyzed by ImageJ software. Percentage of the luminal surface area stained by Oil red-O was determined.

### Human coronary arteries samples preparation

Coronary arteries from the dead donors were used to investigate which cells express ALDH2 and whether ALDH2 expression and activity changed in atherosclerotic vessels. Postmortem human coronary artery samples (n = 6) with wild genotype were collected at autopsy from dead donors in Qilu Hospital, Shandong University. The study complies with the Declaration of Helsinki and was approved by the Institutional Ethics Committee at the institution. After formalin fixation and paraffin embedding, a part of human coronary arteries were serially sectioned at 5μm thickness, and these paraffin sections were used for ALDH2 immunohistochemical staining. And the remaining coronary arteries were frozen at −80°C for western blotting and ALDH2 enzymatic activity detection.

### Cell culture

HUVECs were purchased from the American type culture collection. For the experiments, 4-6 passages of the cells were applied and cultured in HUVEC medium containing 5% fetal bovine serum and 1% endothelial cell growth factors at 37°C. When HUVECs reached 80% confluence, the complete medium was replaced by fresh HUVEC medium containing 0.05% fetal bovine serum. The ALDH2 inhibitor daidzin and ALDH2 activator Alda-1 were applied 2 hours before LPS incubation.

### Extraction of mitochondria

Mitochondria of mice aortas or HUVECs were prepared using a Tissue or Cell Mitochondrial isolation Kits (Beyotime, China) respectively. Briefly, the tissues or HUVECs were washed with 0.01M PBS, centrifuged and homogenized in mitochondrial isolation reagent. The resulting homogenate was centrifuged at 1,000 ×g for 10 min and 3,500 ×g for 10 min at 4°C. The resulting pellet contained the mitochondria. The mitochondria were disrupted, centrifuged at 12,000 ×g for 10 min at 4°C, and the supernatant was frozen at −80°C.

### Measurement of ALDH2 activity

The activity of the mitochondrial ALDH2 was measured at room temperature in 50 mM sodium pyrophosphate (pH = 9.5) containing 2.5 mM NAD^+^, 10 mM acetaldehyde and 50 μg of protein. Acetaldehyde as the substrate of ALDH2 was oxidized to acetic acid, whereas NAD^+^ was reduced to NADH. Production of NADH was determined by spectrophotometric absorbance at 340 nm. ALDH2 activity was expressed as nmol NADH/min/mg protein.

### Western blot analysis

The mice carotid tissues, human coronary arteries or cell lysates of the same protein content (assayed by the BCA method; Bio-Rad, CA, USA) were prepared. Proteins were separated by 10% or 15% SDS-PAGE and transferred to nitrocellulose membranes. The membranes were blocked for 2 hours in a solution of 5% (wt/vol.) milk and then incubated overnight at 4°C with antibodies followed by horseradish peroxidase-conjugated rabbit anti-goat (1:5,000) or goat anti-rabbit IgG (1:5,000 or 1:10,000) for 2 hours. The bands were identified by a standard enhanced chemiluminescence method. ImageJ Software was used to quantitate the intensity of the bands.

### Statistical analysis

All the data were expressed as the mean±SEM. All data were assessed by student t test or one-way ANOVA followed by Student-Newman-Keuls post-hoc analysis. P < 0.05 was considered statistically significant.

## SUPPLEMENTARY MATERIAL FIGURES AND TABLES


