# Supplementary material for: Aldehyde dehydrogenase 2 inhibits inflammatory response and regulates atherosclerotic plaque
Source: Oncotarget. 2016 May 15;7(24):35562–76. doi: 10.18632/oncotarget.9384 (PMC5094945; doi:10.18632/oncotarget.9384)
Supplement: Supplementary file 1 [file oncotarget-07-35562-s001.pdf]

Aldehyde dehydrogenase 2 inhibits inflammatory response and regulates atherosclerotic plaque

Supplementary Material

This file includes:

Supplementary Figure S1: LPS induced MCP-1 and ICAM-1 expression in HUVECs.

Supplementary Figure S2: The effects of Daidzin and Alda-1 on ALDH2 acticity.

Supplementary Table S1: Body weight in three groups of mice.

Supplementary Table S2: Serum lipid levels in ApoE-/- mice.

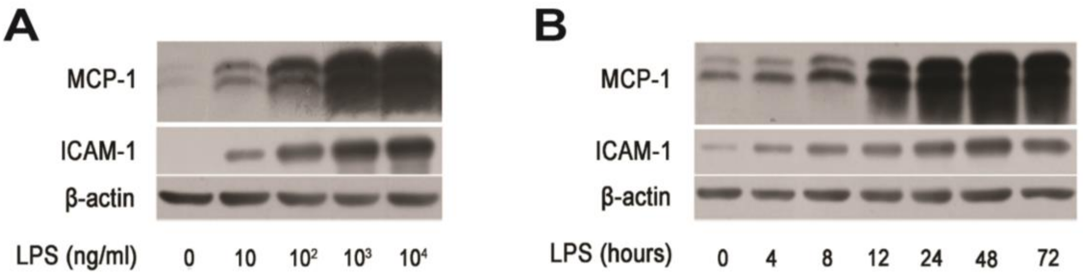

Supplementary Figure S1. LPS induced MCP-1 and ICAM-1 expression in HUVECs. Western blot analysis for LPS-induced concentration-dependent **(A)** and time-dependent **(B)** MCP-1 and ICAM-1 expression changes in HUVECs.

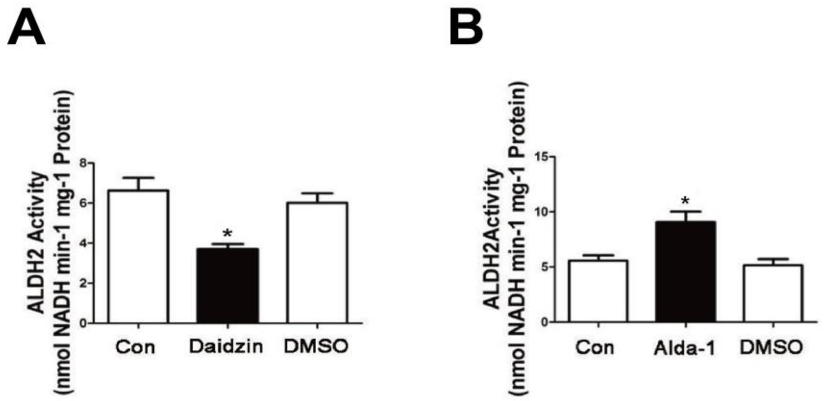

Supplementary Figure S2: The effects of Daidzin and Alda-1 on ALDH2 acticity.

Histograms of effects of 25μM Daidzin **(A)** and 20μM Alda-1 **(B)** on ALDH2 activity in HUVECs. Values are expressed as means±SEM from three independent experiments. *: P<0.05 vs control.

Supplementary Table S1: Body weight in three groups of mice.

			End of 12 weeks (n=58)		
	Baseline (n=60)	End of 8th weeks (n=60)	Lv-GFP group (n=20)	Lv-ALDH2 -overexpression group (n=19)	Lv-ALDH2 -RNAi group (n=19)
Body weight (g)	20.9±0.8	30.1±2.0	32.0±1.9	31.8±2.0	31.3±1.6

Data were displayed as mean±SEM.

Supplementary Table S2: Serum lipid levels in ApoE-/- mice.

	TC (mmol/L)	TG (mmol/L)	HDL-C (mmol/L)	LDL-C (mmol/L)
Lv-GFP group	16.66±1.47	0.66±0.13	1.74±0.15	8.22±0.86
Lv-ALDH2-overexpression group	16.79±1.44	0.62±0.14	1.81±0.14	8.72±0.88
Lv-ALDH2-RNAi group	15.94±1.25	0.70±0.14	1.72±0.15	8.80±0.80

ApoE-/- mice were treated with a high-fat diet (15% cocoa butter and 0.25% cholesterol), as described in the methods section. Data were showed as mean±SEM. TC: total cholesterol; TG: triglyceride
